# Advancing Metastatic Spine Tumor Research: A Review of AO Spine Knowledge Forum Tumor’s Scientific Contributions Derived From the EPOSO Network, 2014-2024

**DOI:** 10.1177/21925682251326515

**Published:** 2025-03-12

**Authors:** Joost P.H.J. Rutges, Scott L. Zuckerman, Paul M. Arnold, Chetan Bettegowda, Stefano Boriani, Michelle J. Clarke, Michael G. Fehlings, Ziya L. Gokaslan, Aron Lazary, Laurence D. Rhines, Arjun Sahgal, Daniel M. Sciubba, James M. Schuster, Michael H. Weber, Ilya Laufer, Charles G. Fisher, on the behalf of the AO Spine Knowledge Forum Tumor

**Affiliations:** 1 Department of Orthopedics and Sports Medicine, 6993 Erasmus Medical Center, Rotterdam, The Netherlands; 2 Department of Neurological Surgery, 12328 Vanderbilt University Medical Center, Nashville, TN, USA; 3 Department of Orthopedic Surgery, 12328 Vanderbilt University Medical Center, Nashville, TN, USA; 4 Department of Neurosurgery, Carle Foundation Hospital, Urbana, IL, USA; 5 Department of Neurosurgery, 1466 Johns Hopkins University School of Medicine, Baltimore, MD, USA; 6 46767 IRCCS Istituto Ortopedico Galeazzi, Milano, Italy; 7 Department of Neurosurgery, 6915 Mayo Clinic, Rochester, MN, USA; 8 Division of Neurosurgery and Spine Program, Department of Surgery, University of Toronto, 7989 University Health Network, Toronto, ON, Canada; 9 Department of Neurosurgery, The Warren Alpert Medical School of Brown University, Providence, RI, USA; 10 National Center for Spinal Disorders, Buda Health Center, Budapest, Hungary; 11 Division of Surgery, Department of Neurosurgery, The University of Texas MD Anderson Cancer Centre, Houston, TX, USA; 12 Department of Radiation Oncology, Sunnybrook Health Sciences Centre, 71545 University of Toronto, Toronto, ON, Canada; 13 Department of Neurosurgery, Zucker School of Medicine at Hofstra, Long Island Jewish Medical Center and North Shore University Hospital, Northwell Health, Manhasset, NY, USA; 14 Department of Neurosurgery, Hospital of the University of Pennsylvania, Philadelphia, PA, USA; 15 Spine Surgery Program, Department of Surgery, McGill University, Montreal, QC, Canada; 16 Department of Neurosurgery, New York University Langone Health, New York, NY, USA; 17 Combined Neurosurgical and Orthopaedic Spine Program, University of British Columbia, Vancouver, BC, Canada

**Keywords:** spine, tumor, metastases, oncology

## Abstract

**Study Design:**

Narrative Review.

**Objectives:**

To summarize the work of the AO Spine Knowledge Forum Tumor, specifically studies from the Epidemiology, Process and Outcomes in Spine Oncology (EPOSO) study.

**Methods:**

A narrative review of all published manuscripts from the EPOSO study was undertaken. EPOSO represents a multicenter, prospective registry effort across 10 North American and European sites to enroll patients with metastatic disease of the spine.

**Results:**

The current review summarized all studies from the EPOSO network, divided into the following five sections: (1) quality of life and satisfaction, (2) overall survival, (3) spinal instability, (4) neurologic outcome in patients with metastatic epidural spinal cord compression or radicular pain, and (5) patient and tumor-specific factors. Several important findings were elucidated. Patient evaluation should include SINS, nutritional status, severity and duration of neurologic deficit, extent of metastatic tumor burden, and differentiation of axial from radicular pain. Moreover, SOSGOQ2.0 serves as a useful and validated instrument for patient-reported outcome instrument. Despite the palliative nature of metastatic spine surgery, clear improvement in quality-of-life is seen. Even in patients with short-survival, the remaining weeks and months of life result in improved quality-of-life. Metastatic spine surgery often improves neurologic function, potentially enhancing survival through increased performance status.

**Conclusions:**

Several noteworthy results have come from the EPOSO network, highlighting important trends in metastatic spine care. The AO Spine Knowledge Forum Tumor has helped advancing metastatic spine tumor research as well as ensure these new findings reach and benefit clinicians and their patients.

## Introduction

The AO Spine Knowledge Forum Tumor has been at the forefront of research in spinal oncology over the past decade. From 2014-2024, this collaborative group has made significant contributions to the field of spinal oncology, publishing numerous high-impact studies that have advanced our understanding of spinal metastases and their management.

An analysis of the bibliometric data (July 2024) from the AO Spine Knowledge Forum Tumor reveals a consistent and impactful publication record. The forum’s research has been published in journals with impact factors ranging from 1.6 to 51.1, demonstrating both breadth and depth in its scientific contributions. Notably, there has been a trend towards increased open access publication in recent years, with 17 out of the 33 articles being freely accessible. This shift reflects a commitment to wider dissemination of knowledge in the field. Citation metrics indicate a strong influence on the scientific community, with several papers accruing over 50 citations. The most cited paper, published in 2016, has 65 citations, underscoring its significance in the field.^1^ More recent publications obviously have less time to accumulate citations, which should be considered when interpreting these metrics. This review aims to synthesize the key findings and trends in the forum’s scientific output during this period from the Epidemiology, Process and Outcomes in Spine Oncology (EPOSO) network.^2^

EPOSO was both a network for multicenter prospective cohort studies in patients with metastatic disease of the spine,^2^ as well as a registry. The compilation of published studies from EPOSO has produced several important findings for clinicians to utilize in their clinical practice. We summarize the research conducted by the AO Spine Knowledge Forum Tumor encompassing the EPOSO network, throughout the last decade, divided into the following five sections: (1) quality of life and satisfaction, (2) overall survival, (3) spinal instability, (4) neurologic outcome in patients with metastatic epidural spinal cord compression, and (5) patient and tumor-specific factors.

## The Epidemiology, Process and Outcomes of Spine Oncology (EPOSO) Study

Metastatic spine disease represents a growing health care challenge, as advancements in oncology extend patient survival, making the spine the most common site for metastases. This shift necessitates focused research to optimize health-related quality of life (HRQOL) for affected individuals. EPOSO addresses this need through a comprehensive, multicenter prospective clinical registry. EPOSO aims to collect a wide range of data on consecutively treated patients with metastatic spine tumors, including patient characteristics, diagnostic information, treatment variables, and both disease-specific and generic HRQOL measures.^2^ By systematically gathering this diverse dataset, EPOSO provides a robust foundation for improving the understanding and management of metastatic spine disease, ultimately contributing to enhanced patient care and outcomes.

The significance of EPOSO lies in its potential to provide comprehensive data on an unprecedented number of metastatic spine tumors, allowing for the determination of many previously hypothesized variables on outcome. Fisher et al emphasize that this multi-center framework will not only collect HRQOL data but also develop a new spine oncology-specific outcome instrument.^2^ The study aimed to improve the quality of spine oncology care globally by generating specific, clinically relevant answers to management questions and establishing benchmarks for comparison among centers.

The primary objectives of EPOSO were 3-fold: (1) to determine the validity and reliability of a specific outcomes questionnaire for patients with metastatic spine disease; (2) to validate the Spine Instability Neoplastic Score (SINS) Classification as a tool for predicting spine stability in metastatic disease; and (3) to determine the effectiveness of surgery vs radiotherapy for treating impending instability secondary to metastatic spine disease. Additionally, de-identified data may be used for further research purposes, including merging with other cohorts like the Metastatic Tumor Research and Outcomes Network **(**MTRON).^3^

The study was conducted across up to 10 clinical sites, primarily sites in North America and Europe. Inclusion criteria encompass patients aged 18-75 years with a diagnosis of metastatic tumor of the spine, able to provide informed consent and read/write in the local language at an elementary level. Exclusion criteria include primary cancer sites in the central nervous system or spine, recent history of substance abuse, or conditions that may preclude accurate evaluation. Between August 2013 and January 2018, a total of 442 patients were included. Of these, 280 patients (64%) received surgical treatment (+/− radiotherapy), while 162 patients (36%) underwent radiotherapy alone. The majority of cases involved a conventional open stabilization with decompression, and tumor debulking.

EPOSO utilizes a comprehensive set of outcome measures, including the Spine Oncology Study Group Outcomes Questionnaire (SOSGOQ), Pain Numeric Rating Scale (NRS), SF-36v2, EQ-5D, Eastern Cooperative Oncology Group (ECOG) Performance Status, and a modified version of the International Standards for Neurological Classification of Spinal Cord Injury (ISNCSCI). Additional data collection includes diagnostic, clinical/imaging, surgical, chemotherapy, radiation therapy, and adverse event information.

The study design follows patients for 24 months or until death, with follow-up assessments at 6, 12-, 26-, 52-, and 104-week post-treatment. Fisher et al emphasize that these follow-ups coincide with standard clinical assessments for spine tumor patients, allowing for integration with routine care.^2^ The study’s observational nature, using standard of care and disease management practices, minimizes study-related medical risks while providing valuable data on patient outcomes and treatment efficacy.

## Summary of Key Studies

### Quality of Life and Satisfaction

Metastatic spine tumor studies on quality of life have provided valuable information on how patients fare in the waning years of life. Answering whether surgery is “worth-it” from the patient perspective have been vital to proving the value of care delivered. The following three studies provide empirical data that surgery leads to improved quality of life and satisfaction.^4-6^ In the first study introducing the Spine Oncology Study Group Questionnaire 2.0 (SOSGOQ2.0), Versteeg and colleagues^6^ sought to validate the novel questionnaire. In 153 patients undergoing metastatic spine surgery with quality-of-life data at 12 weeks, the SOSGOQ2.0 demonstrated strong correlation with SF-36 and discriminated well between different patient groups with good reliability. The authors concluded that the novel SOSGO2.0 questionnaire was a reliable and valid way to evaluate patients with spinal metastases. Most recently in 2024, Barzilai et al^4^ reported initial quality-of-life results of the EPOSO study in 280 patients treated from 2013-18 across the 10 involved centers. With a median overall survival rate of 501 days, significant and durable quality of life improvements were seen at 6-week, 12-week, 26-week, 1-year, and 2-year across several patient-reported outcome questionnaires. Specific to satisfaction in a cohort of 219 treated with surgery and 131 treated with radiation only, Versteeg et al^5^ reported that 84% of patients treated with surgery were satisfied whereas 77% treated with radiation alone were satisfied. Not surprisingly, satisfaction after surgery was associated with improved pain reduction, physical function, mental health, social function, leg function, and EQ-5D. Dissatisfaction after either surgery or radiation was associated with lower baseline strength, function, and social function (Table 1).

**Table 1.** Summary of Key Massages From the AO Spine Knowledge Forum Tumor’s Publications Derived From the EPOSO Network, 2014-2024.

Subject	Key Massages	Publication
Quality of life and satisfaction	In patients undergoing metastatic spine surgery, significant and durable improvements in quality-of-life are seen up to 2-year postoperative	Barzilai et al. 2024^4^
	Patients are generally satisfied after metastatic spine surgery, yet this satisfaction is associated with improved pain, physical function, and mental/social health	Versteeg et al. 2019^5^
	SOSGOQ2.0 is a validated and novel questionnaire to use in the care of metastatic spine patients that is reliable and valid	Versteeg et al. 2018^6^
Overall survival	Patients with oligometastatic disease undergoing metastatic spine surgery generally have longer survival than those with polymetastatic disease	Barzilai et al. 2019^7^
	Even in patients with short survival after metastatic spine surgery, significant quality-of--life improvements are seen that are not significantly different from patients with longer survival	Dea et al. 2020^8^
Spinal instability	Surgical intervention for potentially unstable spinal metastases (SINS 7-12) leads to significant and durable improvements in pain and HRQOL compared to radiotherapy alone	Versteeg et al. 2021^10^
	SINS is a valuable tool for assessing spinal instability, with higher scores correlating with worse pain and physical function at baseline	Versteeg et al. 2021^10^
	Mechanical pain, as assessed by SINS, is strongly associated with PROs and may be a key factor in predicting post-treatment improvements	Versteeg et al 2023^11^
Neurologic outcome in patients with metastatic epidural spinal cord compression or radicular pain	Neurologic deficits in MESCC patients correlate with decreased HRQOL and decreased survival, but surgical treatment can improve or stabilize neurologic function	Barzilai et al. 2019^12^
	Preoperative steroid use in MESCC patients results in neurological stabilization or improvement in 50% of cases but is associated with a high rate of postoperative adverse events	Versteeg et al 2022^13^
	Patients who respond positively to steroid treatment may have a better prognosis, highlighting the potential value of this intervention in select cases	Versteeg et al 2022^13^
	Patients with radicular pain from metastatic spine disease experience greater magnitude of improvement following treatment compared to those with axial pain alone	Dea et al 2024^14^
Patient and tumor-specific factors	Patient-specific factors such as tumor location (sacral, cervical) and gender significantly impact treatment outcomes in metastatic spine disease	Charest-Morin 2019^15^ Bond 2020^16^ Goodwin 2023^17^
	Careful patient selection and multidisciplinary approach are crucial for optimal outcomes, considering factors like spinal instability, baseline pain, and HRQOL scores	

#### Key Messages

• In patients undergoing metastatic spine surgery, significant and durable improvements in quality-of-life are seen up to 2-year postoperatively.
• Patients are generally satisfied after metastatic spine surgery, yet this satisfaction is associated with improved pain, physical function, and mental/social health.
• SOSGOQ2.0 is a validated and novel questionnaire to use in the care of metastatic spine patients that is reliable and valid.

### Overall Survival

Though overall survival may not be directly under the spine surgeon’s control when treating metastatic disease, several studies have shown that survival improves with separation surgery and postoperative stereotactic radiotherapy. In a study comparing 215 patients with oligometastatic disease (≤5 metastases) to 178 patients with polymetastatic disease (>5 metatases), Barzilai et al^7^ reported a significant survival advantage at 3 months and 6 months in the oligometastatic group. Both groups experienced significant improvements in quality-of-life, but the survival advantage was seen most in the oligometastatic group. In a second study addressing survival, Dea et al^8^ investigated whether patients with short survival (<3 months) or longer survival (>3 months) had different quality-of-life after surgery for metastatic disease, with the primary outcome being SOSGOQ2.0 at 6-week postoperative. Of 253 patients undergoing metastatic spine surgery, 40 died within the first 3 months vs 213 patients surviving more than 3 months. Adjusting for baseline performance status, the SOSGOQ2.0 scores at 6 weeks were not significantly different between groups. Moreover, no difference in patient satisfaction at 6 weeks was seen regarding treatment between groups. Interestingly, even despite an increased complication rate among the low survival group (58% vs 39%), the short survival group still had similar SOSGOQ2.0 scores. Looking deeper at all the quality-of-life metrics – NRS-Pain, EQ-5D, SF-36-PCS/MCS, and SOSGOQ2.0, there were some differences worth noting. NRS-Pain scores at 6-week and EQ-5D scores at 12-week were worse for the short survival group. Though most of the long survival group had better scores than the short survival group, the mean scores and group differences were not significantly different. Overall, in patients with an unfortunately low survival, there seemed to still be a benefit from surgical intervention (Table 1).

#### Key Messages

• Patients with oligometastatic disease undergoing metastatic spine surgery generally have longer survival than those with polymetastatic disease.
• Even in patients with short survival after metastatic spine surgery, significant quality-of--life improvements are seen that are not significantly different from patients with longer survival.

### Spinal Instability

Spinal instability is a critical concern for physicians treating patients with metastatic spine disease. The Spinal Instability Neoplastic Score (SINS), developed in 2010 by the Spine Oncology Study Group (the predecessor to AO Spine Knowledge Forum Tumor), has standardized the assessment of spinal instability in cancer patients, improving communication among medical professionals and reporting in spinal neoplastic literature.^9^ Two recent studies have provided valuable insights into the use of SINS and its relationship with patient outcomes. Versteeg et al^10^ investigated outcomes in patients with potentially unstable (SINS 7-12) spinal metastases using the EPOSO network. Among 220 patients, 136 underwent surgery, with or without radiotherapy, while 84 received radiotherapy alone. After controlling for baseline differences, the surgical group showed significant improvements in pain and HRQOL measures up to 52 weeks post-treatment, while the radiotherapy-alone group only demonstrated pain improvement at 12 weeks. In a follow-up study, Versteeg et al^11^ examined the correlation between SINS, its components, and patient-reported outcomes (PROs) in 307 patients. They found moderate correlations between total SINS and baseline SOSGOQ2.0 total score (r = 0.47), physical function (r = 0.50), and pain (r = 0.52). Notably, mechanical pain as a single SINS component showed the strongest correlations with PROs, and patients with mechanical pain experienced greater improvements in health-related quality of life post-treatment (Table 1).

#### Key Messages

• Surgical intervention for potentially unstable spinal metastases (SINS 7-12) leads to significant and durable improvements in pain and HRQOL compared to radiotherapy alone. SINS is a valuable tool for assessing spinal instability, with higher scores correlating with worse pain and physical function at baseline.
• Mechanical pain, as assessed by SINS, is strongly associated with PROs and may be a key factor in predicting post-treatment improvements.

### Neurologic Outcome in Patients with Metastatic Epidural Spinal Cord Compression or Radicular Pain

Treating patients with emergent neurologic deficits is perhaps the most important role of the spine surgeon within the larger landscape of oncologic care. Neurologic deficits in patients with metastatic epidural spinal cord compression (MESCC) can significantly impact surgical outcomes and quality of life.^8^ Two recent studies have provided valuable insights into the management and neurologic outcomes. Barzilai et al^12^ investigated the impact of neurologic deficits on surgical outcomes and health-related quality of life (HRQOL) in 239 MESCC patients from the EPOSO registry. At baseline, 61% of patients were ASIA E, 30% ASIA D, and 9% ASIA A-C. Six weeks post-surgery, 49% of ASIA D patients improved to ASIA E, while 54% of ASIA A-C patients showed improvement, with only 18% reaching ASIA E. Better ASIA scores were associated with improved HRQOL measures and longer survival. In a complementary study, Versteeg et al^13^ examined the effectiveness of corticosteroids in managing preoperative neurological deficits in MESCC patients. Among 30 patients who received steroids preoperatively, 50% experienced neurological deterioration despite steroid use, while 30% stabilized and 20% improved. Longer steroid use did not correlate with improved neurological function, and postoperative adverse events occurred in 60% of patients. However, those who stabilized or improved neurologically after steroid use showed a trend towards improved survival at 3-and 24-month post-surgery.

Dea et al^14^ provided further insights into pain outcomes for patients with radicular symptoms in metastatic spine disease. In their study of 284 patients from the EPOSO registry, 45% had radicular pain with or without axial pain. Patients with radicular pain showed greater improvement in numeric pain rating scale (NRS) scores (from 6.7 to 3.4) compared to those with axial pain alone at 3-month follow-up. Surgical intervention resulted in larger improvements for both groups compared to radiotherapy alone. Interestingly, in a subset of 23 patients with radicular weakness and low-grade epidural compression, modest motor improvement was observed, with a mean increase of 2.1 points in the ASIA score at 3 months (Table 1).

#### Key Messages

• Neurologic deficits in MESCC patients correlate with decreased HRQOL and decreased survival, but surgical treatment can improve or stabilize neurologic function.
• Preoperative steroid use in MESCC patients results in neurological stabilization or improvement in 50% of cases but is associated with a high rate of postoperative adverse events.
• Patients who respond positively to steroid treatment may have a better prognosis, highlighting the potential value of this intervention in select cases.
• Patients with radicular pain from metastatic spine disease experience greater magnitude of improvement following treatment compared to those with axial pain alone

### Patient and Tumor-Specific Factors

Patient and tumor-specific factors play a crucial role in the management and outcomes of metastatic spine disease. The EPOSO network has allowed for specific patient and tumor-specific questions to be answered, providing highly practical and implementable data to spine surgeons. Three recent studies have provided valuable insights into the impact of these factors on treatment decisions and patient outcomes. Charest-Morin et al^15^ investigated the management of sacral metastases using data from the EPOSO network. Among 23 patients with symptomatic sacral metastases, 8 underwent surgery ± RT and 15 received RT alone. Despite worse baseline HRQOL and pain in surgical patients, both groups showed improvements in Pain NRS, EQ-5D, SOSGOQv2.0, and SF-36v2 mental component scores. The study highlighted the validity of both surgical and RT options for sacral metastases, emphasizing the importance of multidisciplinary management. In a study focusing on cervical spine metastases, Bond et al^16^ analyzed 55 patients from the EPOSO registry, with 38 undergoing surgical intervention (+radiotherapy) and 17 receiving radiotherapy alone. Surgically treated patients, despite higher baseline instability and pain scores, demonstrated significant improvements in NRS pain, EQ-5D, and SOSGOQ2.0 scores over 6 months. Lastly, Goodwin et al examined gender differences in 390 patients with spinal metastases. Both genders showed improved HRQOL scores, but females demonstrated longer survival and lower complication rates.^17^ Interestingly, when gender-specific cancers were excluded, the significant improvements in HRQOL for females disappeared, suggesting that primary tumor type may influence outcomes (Table 1).

#### Key Messages

• Patient-specific factors such as tumor location (sacral, cervical) and gender significantly impact treatment outcomes in metastatic spine disease.
• Careful patient selection and multidisciplinary approach are crucial for optimal outcomes, considering factors like spinal instability, baseline pain, and HRQOL scores.

## Discussion

**Figure 1.**
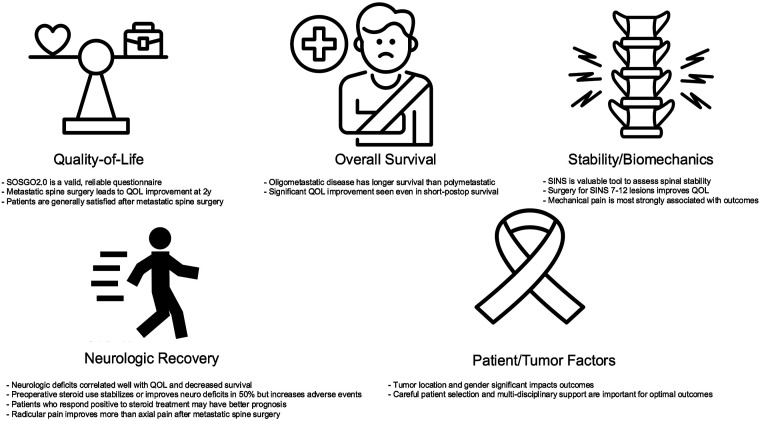
Key messages in the five areas of EPOSO research of quality-of-life, overall survival, stability/biomechanics, neurologic recovery, and patient/tumor factors.

The AO Spine Knowledge Forum Tumor’s scientific output using the EPOSO network over the past decade reflects a comprehensive and multifaceted approach to advancing spinal oncology. The research spans clinical outcomes and patient-reported measures, demonstrating a commitment to addressing the full spectrum of challenges in managing spinal metastases. The compilation of published studies from EPOSO has produced several important findings for clinicians to utilize in their clinical practice. Patients with metastatic spine disease are unique given their palliative setting; a setting requiring a well-informed, shared decision-making process to ensure an optimal treatment plan. Establishing appropriate patient expectations is critical to this process. While most patients improve after surgery, the extent of improvement is variable. Patient evaluation should include SINS, nutritional status, severity and duration of neurologic deficit, extent of metastatic tumor burden, and differentiation of axial from radicular pain. Moreover, SOSGOQ2.0 serves as a useful and validated instrument for PRO measurement^6,18,19^ (Figure 1).

### Key Trends

Several trends are evident in the forum’s work. Despite the palliative nature of metastatic spine surgery, clear improvement in quality-of-life is seen. Even in patients with short-survival, the remaining weeks and months of life result in improved quality-of-life, which is critical for patients and their loved ones. Moreover, metastatic spine surgery often improves neurologic function, potentially enhancing survival through increased performance status. The increasing focus on quality-of-life and patient-reported outcomes aligns with broader trends in oncology towards more patient-centered care. Especially as the cost of health care becomes increasingly important, proving the increased quality-of-life and value of metastatic spine surgery is important to payers and hospitals. Another notable trend is the increasing emphasis on multidisciplinary care. Studies comparing surgical and radiotherapy outcomes, as well as those exploring combined approaches, underscore the importance of collaborative decision-making in managing these complex cases.

### Future

Looking forward, several areas emerge as priorities for future research with the EPOSO network and the AO Spine Knowledge Forum Tumor. These include further refinement of prognostic models, especially those incorporating molecular markers; continued investigation of novel surgical and radiotherapy techniques; and more extensive exploration of quality-of-life outcomes and survivorship issues in the context of improving systemic therapies. Moreover, as we move into the next decade, the challenge will be to translate these research findings into improved clinical outcomes and quality of life for patients with spinal metastases. The trends towards personalized medicine (targeted molecular therapy), minimal invasive surgery, advanced radiotherapy techniques, multidisciplinary care, and patient-centered outcomes are likely to continue shaping the field, with the AO Spine Knowledge Forum Tumor well-positioned to lead these advancements.

However, we must acknowledge that the traditional research paradigm of “present, publish, and proceed” has led to gaps in knowledge translation and clinical implementation. Moving forward, we need to prioritize not just the generation of new knowledge, but also its effective dissemination and integration into clinical practice. This requires a more comprehensive approach where researchers actively plan for and support the implementation of their findings, ensuring that valuable research insights actually translate into improved patient care at the bedside. As a leading organization in spinal oncology research, the AO Spine Knowledge Forum Tumor must take a proactive role in advancing not only the science but also the knowledge translation strategies that ensure research findings effectively reach and benefit practicing clinicians and their patients.
